# Genome-Wide Analysis on Transcriptome and Methylome in Prevention of Mammary Tumor Induced by Early Life Combined Botanicals

**DOI:** 10.3390/cells12010014

**Published:** 2022-12-21

**Authors:** Itika Arora, Shizhao Li, Michael R. Crowley, Yuanyuan Li, Trygve O. Tollefsbol

**Affiliations:** 1 Department of Biology, University of Alabama at Birmingham, Birmingham, AL 35294, USA; 2 Heflin Center for Genomic Science, Department of Genetics, University of Alabama at Birmingham, Birmingham, AL 35294, USA; 3 Department of Nutrition and Food Science, University of Maryland, College Park, MD 20742, USA; 4 O’Neal Comprehensive Cancer Center, University of Alabama at Birmingham, Birmingham, AL 35294, USA; 5 Integrative Center for Aging Research, University of Alabama at Birmingham, Birmingham, AL 35294, USA; 6 Nutrition Obesity Research Center, University of Alabama at Birmingham, Birmingham, AL 35294, USA; 7 Comprehensive Diabetes Center, University of Alabama at Birmingham, Birmingham, AL 35294, USA

**Keywords:** breast cancer, prevention, transgenic mice, broccoli sprouts, green tea polyphenols, RNA-seq, RRBS, epigenetics

## Abstract

Breast cancer (BC) is the most common malignancy and the second leading cause of cancer death among women in the United States. The consumption of natural dietary components such as broccoli sprouts (BSp) and green tea polyphenols (GTPs) has demonstrated exciting potential in reducing the risk of BC through the regulation of epigenetic mechanisms. However, little is known about their impacts on reversing epigenomic aberrations that are centrally involved in the initiation and progression of BC. Previously, we have determined the efficacy of combined BSp and GTPs treatment on the inhibition of the growth of a mammary tumor in a transgenic Her2/neu mouse model. We sought to extend our previous study to identify universal biomarkers that represent common mechanistic changes among different mouse models in response to this dietary regime by including a new transgenic mouse model, C3(1)-SV40 TAg (SV40). As a result, we identified novel target genes that were differentially expressed and methylated in response to dietary botanicals when administered singly (BSp and GTPs) and in combination (BSp + GTPs) in both mouse models. We discovered more differentially expressed and methylated genes in the combination treatment group compared to the singly administered groups. Subsequently, several biological pathways related to epigenetic regulations were identified in response to the combination treatment. Furthermore, when compared to the BSp and GTPs treatment alone, the combinatorial treatment showed a more significant impact on the regulation of the epigenetic modifier activities involved in DNA methylation and histone modifications. Our study provides key insights about the impact of the combined administration of BSp and GTPs on BC using a multi-omics analysis, suggesting a combinatorial approach is more efficacious in preventing and inhibiting BC by impacting key tumor-related genes at transcriptomic and methylomic levels. Our findings could be further extrapolated as a comprehensive source for understanding the epigenetic modifications that are associated with the effects of these dietary botanicals on BC prevention.

## 1. Introduction

Breast cancer (BC) is the most common malignancy and the leading cause of death among women worldwide [1]. The BC incidence and death rate vary based on race and ethnicity. The onset of BC can also be attributed to environmental factors, such as nutrition and diets [2]. A body of evidence implicates the role of bioactive dietary compounds in the prevention and treatment of multiple types of human cancers, including BC [3]. Epidemiological studies have demonstrated that the incidence of BC is relatively lower in Asian women due to the higher consumption of multiple vegetables and fruits [4]. The bioactive dietary components such as the polyphenols in those plants exhibit biological activities involved in various cell signaling, such as inhibiting RTK/RAS and PI3K and inducing p53 signal pathways, contributing to their chemopreventive effects against BC [5]. Previous studies have demonstrated that bioactive diets such as green tea polyphenols (GTPs) that contain a major polyphenol, epigallocatechin-3-gallate (EGCG), are efficacious in preventing and inhibiting various human cancers [5,6,7]. Additionally, cruciferous vegetables such as broccoli sprouts (BSp) that contain the bioactive isothiocyanate, sulforaphane (SFN), play an active role in the prevention of tumorigenesis [8]. 

Cancer progression is closely related to aberrations of genetic modifications and epigenetic alterations. Histone modifications and DNA methylation are the most important epigenetic mechanisms [9]. DNA methylation is carried out by DNA methyltransferases (DNMTs), and the DNA methylation status can vigorously influence transcriptional activities [10]. Histone modifications are key epigenetic events that modulate the chromatin structure and alter accessibility to transcription factors, thereby impacting the transcriptional efficiency. Studies in the past revealed that the profusion and localization of epigenetic landmarks are reactive to various environmental stimuli such as diet-related changes, which can affect gene expression and eventually lead to phenotypic changes [11].

Several mechanisms have been proposed to explain the chemopreventive properties of SFN in BSp, including the induction of apoptosis, cell cycle arrest and the activation of phase I Cytochrome P450s (CYP) enzymes and phase 2 detoxifying enzymes [12,13,14]. SFN has recently received considerable attention due to its ability in the regulation of epigenetic processes by targeting key epigenetic modulators, such as histone deacetylases (HDACs) and DNMTs. Eventually, this could influence gene expression profile changes by affecting epigenetic hallmarks on the local and global scales [5,15,16,17,18]. The EGCG in green tea has also been proposed to have epigenetic effects in the regulation of the gene expression and enzymatic activities of DNMTs and HDACs [6,7,16,17,18]. We have previously shown that the combined administration of EGCG in GTPs and SFN in BSp can synergistically inhibit BC proliferation in vitro and in vivo [16]. We further tested the effects of GTPs and BSp in a transgenic mouse model, Her2/neu, and determined the impact of this combinatorial regimen on an epigenome [8]. To test whether the potent anticancer properties and essential regulatory roles of this novel dietary regimen on an epigenome are ubiquitous, we extended our study to investigate the combined effects of BSp and GTPs on BC in a different transgenic mouse model, SV40, and explored the potential epigenetic mechanisms.

Our results showed that the combined administration of BSp and GTPs showed more prominent effects on BC inhibition by modulating epigenetic profiles. Herein, we performed a correlated analysis between the DNA methylome by reduced representation bisulfite sequencing (RRBS) and RNA transcriptome by RNA sequencing in an SV40 mouse model with the single and combination treatment of BSp and GTPs. Our results showed the cumulative impacts of BSp and GTPs on the transcriptomic and methylomic levels. In comparison with the data from our previous studies in a Her2/neu model, we identified two differentially expressed genes (*Cbl* and *Zfp800*) and several differentially methylated genes that showed overlapped expression or methylation change patterns across both mouse models. Overall, our findings are important by uncovering novel biomarkers in response to this combinatorial dietary regimen that can potentially be used for future breast cancer prevention or therapy.

## 2. Materials and Methods

### 2.1. Mouse Model

The transgenic mouse model, C3(1)-SV40 TAg (FVB-Tg(C3-1-TAg)cJeg/JegJ), was purchased from Jackson Lab (Bar Harbor, ME, USA). These mice are known to develop spontaneous ER(−) mammary tumors due to overexpressed SV40 transgene with a median tumor latency of ~15 wks [19]. The female mice were bred at approximately 10 wks of age to generate sufficient colonies for further investigation. The litters were weaned at 21 days after birth and genotyped by performing a standard PCR analysis on their snipped tails. Mice were housed in the University of Alabama at Birmingham’s Animal Resource facility and further sustained under 12 h light/dark cycle, 24 ± 2 °C temperatures and 50 ± 10% humidity. All animals had free access to food and water. The animal study was reviewed and approved by the Institutional Animal Use and Care Committee of the University of Alabama at Birmingham (IACUC; Animal Project Numbers: 10,088, 20,653 and 20,671).

### 2.2. Dietary Treatment

Female C3(1)-SV40 TAg (SV40) mice were assigned to four groups and administered with the dietary botanicals from prepubescence (3 wks) until termination (20 wks) of the experiment. The control group (N_Control_ = 9) was administered control AIN-93G diet. In the BSp group (N_BSp_ = 7), mice were administered a modified 26% (w/w) BSp diet (TestDiet, St. Louis, MO, USA) as used previously [8]. In the GTPs group, mice (N_GTPs_ = 7) were orally administered with 0.5% GTPs Sunphenon 90D (Sunphenon 90D, Taiyo International, Inc., Minneapolis, MN, USA) in drinking water, which contains > 90% polyphenols. Lastly, in the combination group, mice (N_Combination_ = 7) were fed with a combination BSp and GTPs diet as described above. The BSp and GTPs concentrations incorporated in this study were pharmacologically achievable and also possessed translational potential [8,16,20,21,22]. 

### 2.3. Tumor Collection and Evaluation

Tumor incidence was measured and recorded weekly [23,24]. Additionally, body weight was recorded biweekly. We employed tumor latency as the primary outcome followed by tumor weight due to the spontaneous growth nature of mammary tumor in the transgenic mouse model. Food and water intake were measured at 4, 12 and 20 wks of age. The experiment was terminated at 20 wks when the average tumor diameter of mice in the control group exceeded 1.0 cm. At the end of the investigation, the mice were euthanized by CO_2_. The mammary tumors were excised, weighed and snap-frozen in liquid nitrogen for further analysis. Tumor incidence was determined using the Chi-square test. A two-tailed Student’s t-test was performed to evaluate significant difference between two groups, and one-way independent ANOVA was performed to compare three or more groups. Tukey’s post hoc test was also performed to assess significant differences across the groups. Error bars were the standard error of the mean obtained from experiments. Statistically significant results were represented as ** for *p*-value < 0.01 and * for *p*-value < 0.05. 

### 2.4. DNA and RNA Isolations

Genomic DNA (gDNA) and total RNA were extracted from the same frozen mammary tumor that was used for library construction. The gDNA was extracted using a DNAeasy kit (Qiagen, Valencia, CA, USA) as per the manufacturer’s protocol. DNA concentration and quality were measured using a NanoDrop spectrophotometer (Thermo Fisher Scientific, Waltham, WA, USA) and Qubit dsDNA High sensitivity kit. Subsequently, total RNA was extracted using TRIzol reagent (Sigma-Aldrich, St. Louis, MO, USA) based on the manufacturer’s protocol and RNA concentration was determined using NanoDrop spectrophotometer. RNA integrity numbers (RIN) were evaluated using an RNA Nano bioanalyzer chip, and only the samples with RIN > 7 were retained for sequencing. After the isolation, RNA and DNA were frozen and kept at −80 ℃ until further use. 

### 2.5. Library Construction and Sequencing for RNA-Seq and Reduced Representation Bisulfite Sequencing (RRBS)

Both RNA-seq libraries pair-end reads (75 bp × 2) and RRBS libraries single-end reads were sequenced on Illumina NextSeq 500 at the Heflin Center of Genomic Sciences (University of Alabama at Birmingham, USA). For each mRNA preparation (N_Control_ = 6, N_BSp_ = 7, N_GTPs_ = 3 and N_Combination_ = 3), approximately 45 million sequencing reads were generated per sample, which were considered for library construction. In RRBS libraries, the bisulfite-converted libraries yielded an average of 75 million high-quality 75 bp pair-end reads, indicating that the bisulfite conversion efficiency was greater than 98% overall.

### 2.6. Bioinformatics Pipelines

The RNA-seq data processing began with assessing the read quality across all the control, BSp, GTPs and combination samples using FastQC (v0.11.9). Briefly, the RNA-seq raw fastq files were trimmed and further aligned using a pseudo-aligner Salmon [25] to mouse reference NCBI GRCm39 genome. The aligned reads for all the samples in different treatment groups were used to generate a count matrix over the entire mouse transcriptome (GRCm39). The transcript abundance read estimates generated from GRCm39 across different treatment groups were imported into R (v3.6.3) using tximport [26] and further normalized for sample sequencing depth using an R-based Bioconductor package, DESeq2 [27]. As a result, we determined the normalized expression levels across control, BSp, GTPs and combination treatment groups. Furthermore, the differential expression across control-BSp, control-GTPs and control-combination groups were also analyzed using DESeq2. The transcripts across different treatment groups were considered differentially expressed (DE) if Benjamini–Hochberg false discovery rate (implemented in DESeq2) was ≤0.05 and absolute value for log_2_ Fold change was ≥1.5 [28]. 

For the RRBS data, we utilized the pipeline that integrated the assessment of the read qualities (FastQC, v0.11.9), followed by the trimming process (TrimGalore, v0.4.5, NuGEN diversity trimming), alignment using Bismark (v0.16.34) [29] and differential methylation analysis using MethylKit [30]. Firstly, the RRBS raw fastq files data were trimmed using Trim Galore and then aligned to the mouse reference genome (NCBI GRCm39) using Bismark under default parameter settings. The methylation call files comprised of each CpG site and methylation percentage were generated using bismark_methylation_extractor function in Bismark. The aligned BAM files were further processed and used to generate a CpG profile using diffmeth function in Bismark. The CpG coverage with a minimum of 20 reads across the samples in different treatment groups was used for further analysis. 

### 2.7. Gene Networks, Pathways and Functional Annotation Analyses

Significant genes at the transcriptomic and methylomic levels were used to analyze gene networks, canonical and functional pathways. The identified differentially expressed genes (DEGs) that were used as an input into the STRING database [31] to unravel the protein–protein interaction (PPI) network using ClueGO plugin in Cytoscape (3.6.0) [32], and the identified differentially methylated genes (DMGs) were used as an input to identify biological pathways using Database for Annotation, Visualization and Integrated Discovery (DAVID). These DEGs and DMGs were identified from combination treatment group.

### 2.8. Statistical Analysis and Principal Component Analysis (PCA) 

Principal component analysis (PCA) components were calculated from normalized gene expression data across different treatment groups using the prcomp function in R package stats (v3.6.3). Finally, the contributions of each PCA component were extracted using the “get_eigenvalue” function. For transcriptome analysis, fold change (FC) ≥ 1.5 and *p* ≤ 0.05 were considered as a threshold to select DEGs across the treatment groups (control-BSp, control-GTPs and control-combination). A methylation change ≥ 10% (FDR ≤ 0.05) was considered a threshold for the methylome analysis to identify DMGs. For other statistical computations and analysis, data were presented as mean ± SD, which were further analyzed using a two-tailed Student’s t-test or one-way ANOVA along with Tukey’s post hoc test.

### 2.9. Quantitative Real-Time PCR

Gene expression for specific genes of interest was examined by real-time PCR with SYBR GreenER qPCR Supermix (Invitrogen, Waltham, WA, USA). For PCR arrangement, we used 2 µL of cDNA, 4 µL of iTaq SYBR green from Bio-Rad, 2 µL of nuclease-free water, 1 µL of forward and reverse primers for specific genes of interest with a total volume of 10 µL. Upon the preparation of the samples, the gene expression was assessed in triplicates by PCR using the CFX Connect Real-Time System (Bio-Rad, Hercules, CA, USA). Specific gene primers for DE across SV40 and Her2/neu mouse models were obtained from Integrated DNA Technologies (Coralville, IA, USA) (Table 1). 

### 2.10. Global DNA Methylation, Hydroxymethylation and Histone Methylation Analysis 

DNA extraction from mammary tumors of control, BSp, GTPs and combination treatment groups was described before. The global DNA methylation status was explicitly indicated by the levels of 5-methylcytosine (5-mC) in total DNA and was determined by the MethylFlash Methylated DNA 5-mC Quantification Kit from EpiGentek. In addition, the MethylFlash Hydroxymethylated DNA 5-hmC Quantification Kit (EpiGentek, Farmingdale, NY, USA) was employed to quantify global hydroxymethylation status in total DNA samples. The nuclear protein was extracted to determine overall DNMT and HDAC enzymatic activities using the EpiQuik DNMT Activity/Inhibition Assay Ultra Kit (EpiGentek) and the EpiQuik HDAC Activity/Inhibition Assay Kit (EpiGentek), respectively. Moreover, histone acetylation activities were evaluated using EpiQuik Acetyl Histone3-Lysine9 and Histone3-Lysine27 (H3K9 and H3K27) Assay Kits as per the manufacturer’s guidelines.

## 3. Results

### 3.1. Study Design

**Figure 1:**
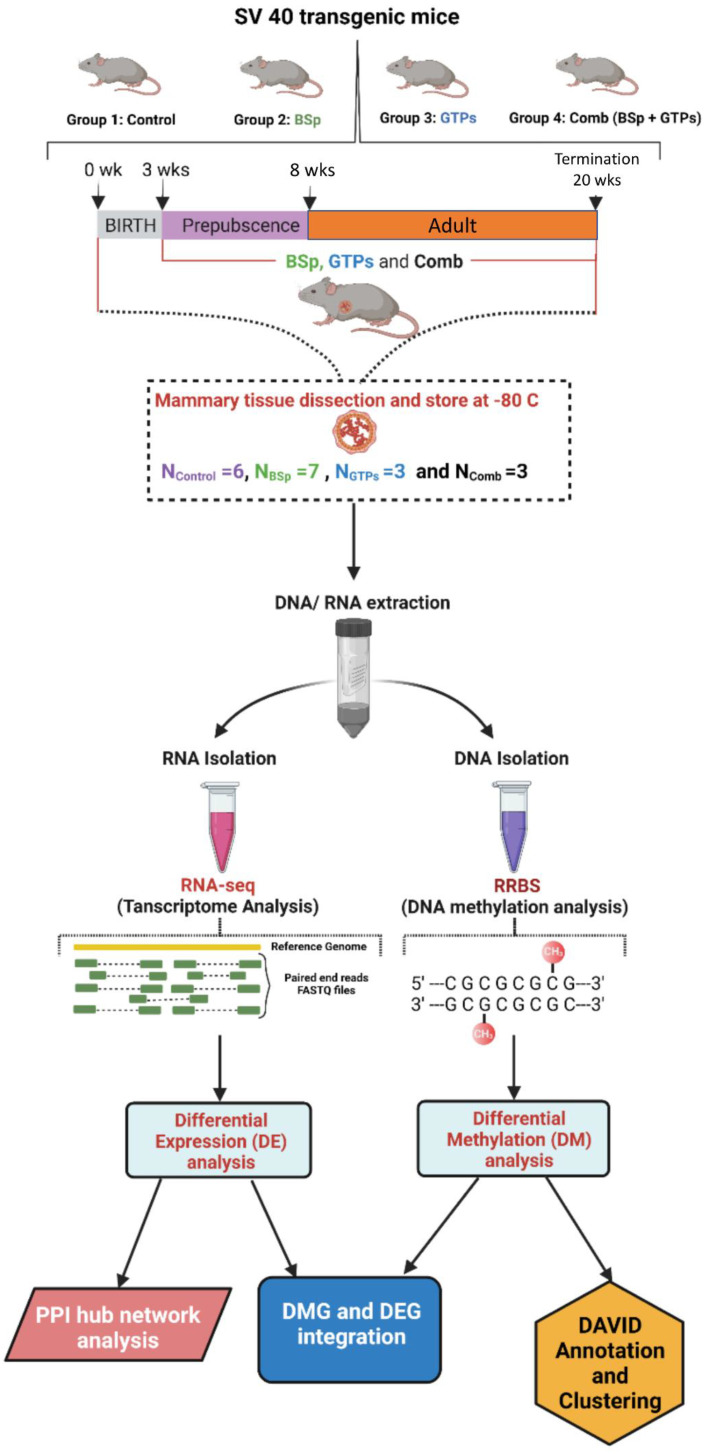
Study Design. An overview of framework demonstrates experimental design and data analysis pipeline. Dietary treatment across different groups (Group 1: control; Group 2: BSp; Group 3: GTPs and Group 4: combination (BSp + GTPs) started at 3 wks of age and mice were euthanized at 20 wks when all mice in the control group developed mammary tumors. RNA and DNA were extracted from the harvested mammary tumors, and then RNA-seq and RRBS libraries were constructed to obtain transcriptome and DNA methylome, respectively. Disease and functional pathways were generated using DAVID, gene ontology (GO) functional annotation based on the DEGs and DMGs, respectively. This figure was created using BioRender (https://biorender.com, accessed on 13 November 2021).

Figure 1 illustrates the overall study design. The harvested tumor samples derived from female SV40 mice for different treatment groups were used for designing the RNA-seq libraries and RRBS methylation libraries. The libraries were multiplexed with unique samples, pooled and sequenced on an Illumina NextSeq 500 platform. Before mapping the libraries to the mouse NCBI GRCm39 genome, the constructed libraries were subjected to FastQC to determine the libraries’ overall quality, thereby identifying adapter and low-quality trimmed reads. Subsequently, the identified DEGs were used to construct the protein–protein interaction (PPI) network using the STRING database and Cytoscape, followed by the network analysis using Cytoscape and a DMGs analysis using the DAVID functional annotation.

### 3.2. Dietary Treatment with BSp, GTPs and Combination Prevented Mammary Tumor Development in Transgenic Mice

We used an SV40 mouse model in this study that can spontaneously develop mammary tumors early in life due to the overexpression of the SV40 oncogene. The dietary concentration for the BSp and GTPs used in this study was formulated as 26% BSp chow diet (*w*/*w*) and 0.5% GTPs in the drinking water, respectively. The concentrations of these diets are physiologically available and demonstrate a practical consumption level by consuming ~2 cups BSp/day or drinking 1–2 cups of green tea for an adult human [8,16,22]. 

**Figure 2:**
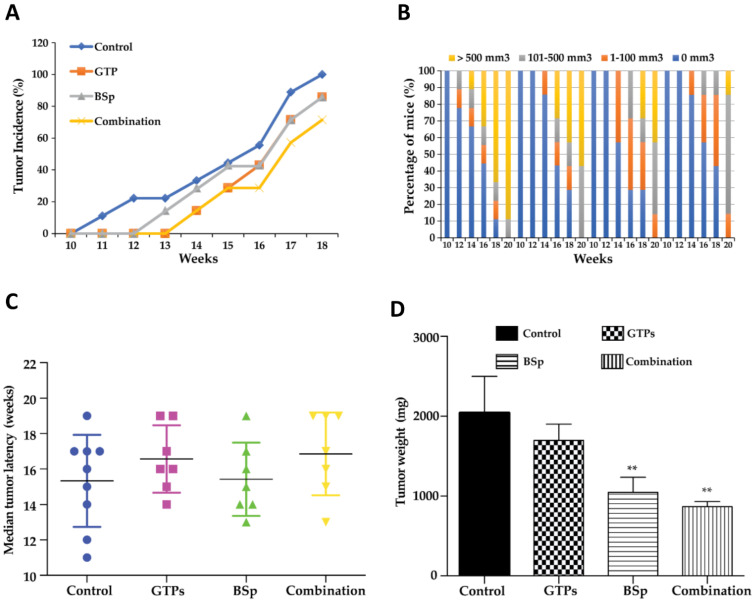
Breast tumor growth in SV40 mice exposed to different dietary botanicals. SV40 mice were administered with control diet, 26% BSp diet, 0.5% GTPs in drinking water or BSp and GTPs in combination (BSp + GTPs) upon weaning at 3 wks of age. Dietary treatment was maintained throughout the study until the termination of the experiment, and mice across each treatment group (control, BSp, GTPs and combination) were evaluated for tumor growth weekly. (**A**) Tumor incidence was measured in percentage over the whole population. (**B**) Tumor growth volume was measured in rate across the entire population. (**C**) Median tumor latency between BSp, GTPs and the combination treatment group. (**D**) Average tumor weight between BSp, GTPs and the combination treatment group. Columns represent mean; bars, standard error; ** *p*-value < 0.01, significantly different from the control group.

We investigated the effects of the BSp, GTPs and combination (BSp + GTPs) treatments on the tumor development in the SV40 transgenic mice. Our results showed that both the single and combinatorial treatment of GTPs and BSp led to the suppression of tumor growth (Figure 2). However, the combinatorial treatment resulted in a more effective inhibition of breast tumor growth via a decreasing tumor incidence and tumor volume in the SV40 transgenic mice (Figure 2A,B). Although the combinatorial treatment extended the tumor latency in the SV40 mice, this effect was not statistically significant (Figure 2C). We found that the combination group affected the tumor development during the rapid progression stage, leading to a significant inhibition in the tumor weight (Figure 2D). Our results demonstrated that dietary exposure to the combined treatment with BSp and GTPs can lead to a prominent inhibition of BC in SV40 mice, which is consistent with our previous studies in a different transgenic Her2/neu mouse model [8]. Based on these results, we conducted the relevant genome-wide analysis in the SV40 model and compared the results at the transcriptomic and methylomic levels with those in a Her2/neu model. 

### 3.3. Informatics Pipeline and Overall Quality Control (QC) of RNA-Seq Transcriptomic Data and RRBS DNA Methylomic Data

To study the combinatorial effects of BSp and GTPs on the epigenomic and transcriptomic changes in comparison to the BSp and GTPs treatment alone, we constructed 19 (N_Ctrl_ = 6, N_BSp_ = 7, N_GTPs_ = 3 and N_Combination_ = 3) libraries for the RNA-seq and RRBS analysis, respectively. Figure 1 demonstrates the study design, ranging from the dietary treatment and further extending the study to a downstream analysis. The RNA-seq library size was distributed within 250–500 bp and the peak was around 300 bp. The fragment size of the RRBS libraries was between 200 and 500 bp with the peak around 275 bp. Overall, we generated ~764 million reads (75 bp × 2) of pair-end transcriptomic data (N =19) per RNA-seq samples and ~800 million reads (75 bp) of single-end DNA methylome (N =19) data per RRBS sample. Eventually, we obtained high-quality reads from both the RNA-seq and RRBS data. We aligned the transcriptomic and methylomic data to the NCBI mouse GRCm39 genome. On average, ~85.05 % (N =19) of the reads were uniquely aligned to the genome.

### 3.4. Global Transcriptomic Changes Induced by Dietary Administration of BSp and GTPs Singly and in Combination 

According to our current research and previous publications [8], the combined BSp and GTPs showed the greatest preventive and inhibitory effects on BC compared to these two compounds administered separately in a spontaneous Her2/neu mouse model. We further performed RNA-seq analyses in the mammary tumors of an SV40 mouse model across different treatment groups (control, BSp, GTPs and combination) to elucidate the global gene expression changes in the combination treatment in comparison with the individually administered BSp or GTPs [16]. The RNA-seq data were first transformed using linear modeling and eventually all the samples were outlined by generating a boxplot (Supplementary Figure S1). A histogram was generated to assess the distribution of the samples, thereby following a normal distribution (Supplementary Figure S2). A two-dimensional plot was created to observe the samples’ overall spatial arrangements across the different treatment groups by conducting unsupervised learning on the gene expression profiles (Supplementary Figure S3). As a result, the samples overlapped both PCAs (PC1 vs. PC2). Among the total identified 14,766 transcripts, 193 genes were DE by the BSp treatment, out of which 119 (61.66%) genes were upregulated and 74 (38.34%) were down-regulated. Additionally, 49 genes were DE in the GTPs treatment, wherein 30 (61.22%) genes were upregulated and 19 (38.78%) were down-regulated. In comparison to the singly administered groups, the combination treatment showed a higher number of DE genes (N_DE genes_= 250), amongst which 225 (90%) genes were upregulated and 25 (10%) were down-regulated using a 5% false discovery rate (FDR) and a fold-change (log_2_ FC) cutoff greater than 2 (Table 2).

**Figure 3:**
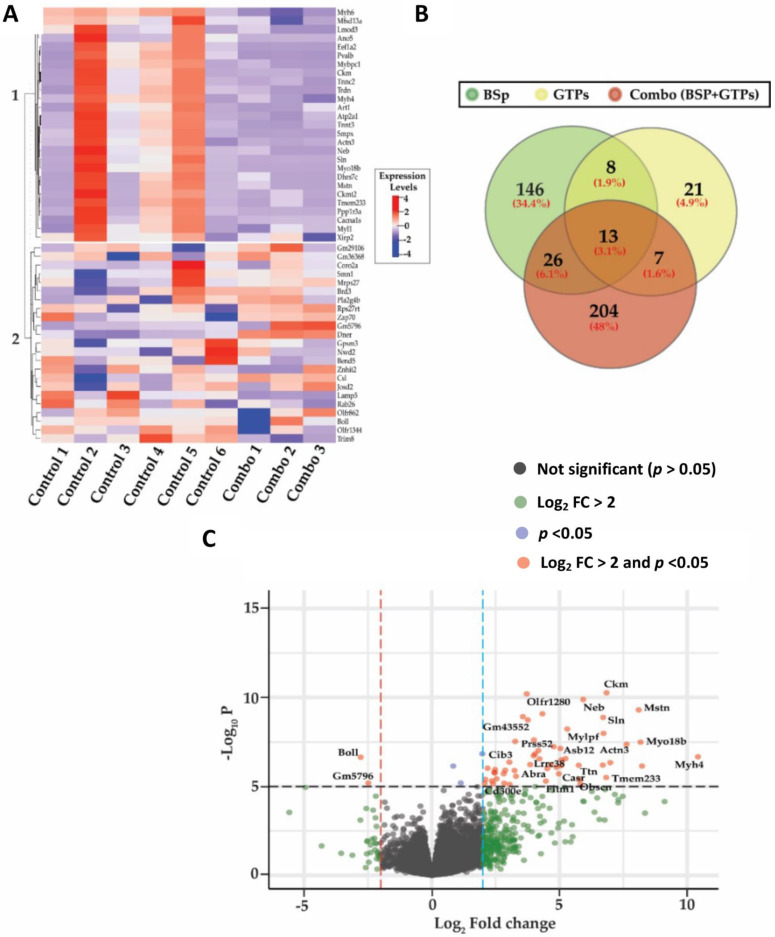
Transcriptomic-level changes across BSp, GTPs and combination (BSp + GTPs) treatment groups. (**A**) Heatmap represents top 50 DEGs in the combination (BSp + GTPs) treatment group, where each row corresponds to differentially expressed transcripts and each column represents sample replicates in control (N_Control_ = 6) and the combination (N_Combination_ = 3) group across two heatmap clusters. Blue indicates lower expression levels, and red denotes higher expression levels. (**B**) Venn diagram exhibits a total number of unique and intersecting differentially expressed genes in BSp, GTPs and combination (BSp + GTPs) treatment groups. (**C**) Volcano plot shows distribution of identified transcripts in combination treatment group. The red dots represent the most significantly changed DEGs with Log_2_ fold change (FC) > 2 and *p* value < 0.05.

To better understand the transcriptional profile changes across the different dietary groups, we generated a heatmap for the top 50 DEGs, where the rows correspond to the DEGs, and the columns correspond to the biological replicates in the control and combination treatment groups (Figure 3A). In addition, we included top 20 up- or down-regulated DEGs in response to combination treatment ranked by fold change (Table 3). We also generated a heatmap for the top 50 DEGs between the control and BSp treatment groups (Supplementary Figure S4A) and 49 DEGs in the control and GTPs treatment groups (Supplementary Figure S4B). Consequently, only the unique transcripts in the combination treatment group were used for the downstream analysis (Figure 3B). The unique DEGs in the combination group were further visualized using a volcano plot to better understand the expression-level changes (Figure 3C). A comprehensive list of all the DE genes across the BSp, GTPs and combination (BSp + GTPs) treatment groups are displayed in Supplementary File S1: Tables S1–S6.

**Figure 4:**
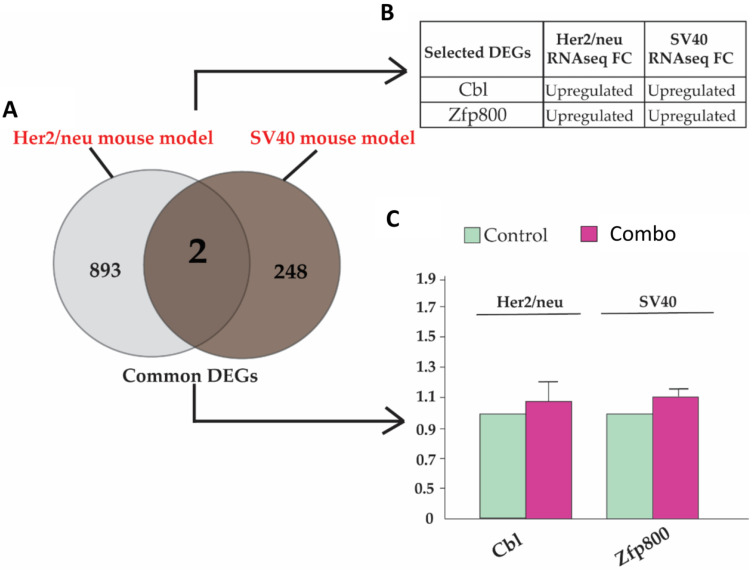
DEGs in the SV40 and Her2/neu mouse models. (**A**) Venn diagram of DE genes between the SV40 and Her2/neu mouse models. A total of 895 and 250 DEGs were identified in Her2/neu (gray) and SV40 (brown) mouse models, respectively. Among these, two DEGs were identified in both strains. (**B**) The table shows gene expression patterns of these common DEGs across two different mouse models. (**C**) qRT-PCR validation of gene expression of *Cbl* and *Zfp800* in both mouse models. Values are expressed as each gene normalized on the expression of respective control (mean ± SE in three replicates).

Furthermore, we compared the RNA-seq results in the SV40 model with our previous study in the Her2/neu model [8]. Interestingly, we identified two DE genes (*Cbl* and *Zfp800*) in response to the combination treatment in both the SV40 and Her2/neu mouse models (Figure 4A). These identical gene expression patterns demonstrate that the combination treatment group may regulate similar transcriptional-level changes in these two specific genes and their related pathways in both mouse models (Figure 4B). Additionally, the qRT-PCR detection on the gene expression in the mammary tumors from both models further confirmed the increased expression patterns, which were consistent with the relevant results from the RNA-seq (Figure 4C). However, this increment was not significant, which may be due to the small sample size or the lower sensitivity of the qPCR capability. Unlike the combination treatment group, the BSp or GTPs treatment did not show overlapping DEs at the transcriptomic level in both mouse models. Many studies from the previous literature have reported that these genes are important tumor-related genes. For instance, a study provided a comprehensive description of the cancer-related KRAB-ZNF (Kruppel-associated box domain zinc finger) gene family using the Cancer Genome Atlas pan-cancer Database. As a result, 16 KRAB-ZNF clusters were identified to be upregulated across different cancers, such as those of the lung and BC [33]. Another study also identified that *Cbl* is highly expressed in BC and significantly inhibits the transforming growth factor-β (TGF-β) tumor-suppressive activity [34]. Our results showed that the combination (BSp + GTPs) treatment can impose more significant effects on expression-level changes because we identified a greater number of DE genes in comparison to BSp and GTPs administered alone in both the SV40 and Her2/neu mouse models. This significant gene expression-level change by the combination treatment may contribute to more efficacious chemopreventive effects on BC compared to any single treatment.

### 3.5. Construction of Protein–Protein Interaction (PPI) Hub Networks 

**Figure 5:**
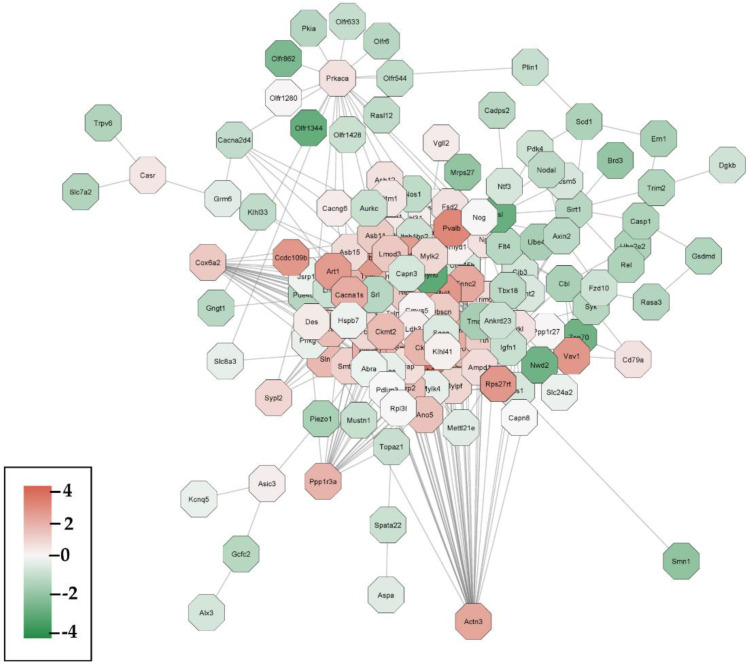
The enlarged regulatory relational network was generated using Cytoscape. The color of the nodes denotes the direction of expression change; red nodes indicate the upregulated genes, while green nodes stand for the down-regulated genes. The color scale measures the expression changes between the control and the combination (BSp + GTPs) treatment group.

The combination treatment-induced DEGs in the SV40 mice were uploaded into the STRING (v11) [31] database using a confidence score of 0.4 in order to avoid false positives. We further generated a unique PPI network using the ClueGO plugin in the cytoscape [32]. Based on the neighbor extension method, we constructed a PPI network that consists of 250 DEGs by the combination treatment, leading to an overall 1942 regulatory relationships. These candidate genes with specific expression-level changes (using the eBayes moderated *t*-test *p*-value ≤ 0.05) were color coded with red (upregulated genes) and green (down-regulated genes) as shown in Figure 5. As a result, we identified that a majority of the genes being upregulated formed a stronger hub network in comparison to the down-regulated genes. 

### 3.6. Genome-Wide DNA Methylation Changes in Response to Combination Treatment

To further elucidate the global DNA methylation changes across the different dietary treatment groups, we applied RRBS analyses in the mammary tumor samples of the SV40 mice. A total of nineteen single-end libraries (N_Ctrl_ = 6, N_BSp_ = 7, N_GTPs_ = 3 and N_Combination_ = 3) were designed. Each of these libraries produced a minimum of seven Gb clean reads, which were sequenced and aligned to the reads of the mouse reference NCBI GRCm39 genome using Bismark [29]. The reads of the individual samples were mapped to the reference genome within each group, which were further used for the downstream analysis (Supplementary File S2: Table S7). 

**Figure 6:**
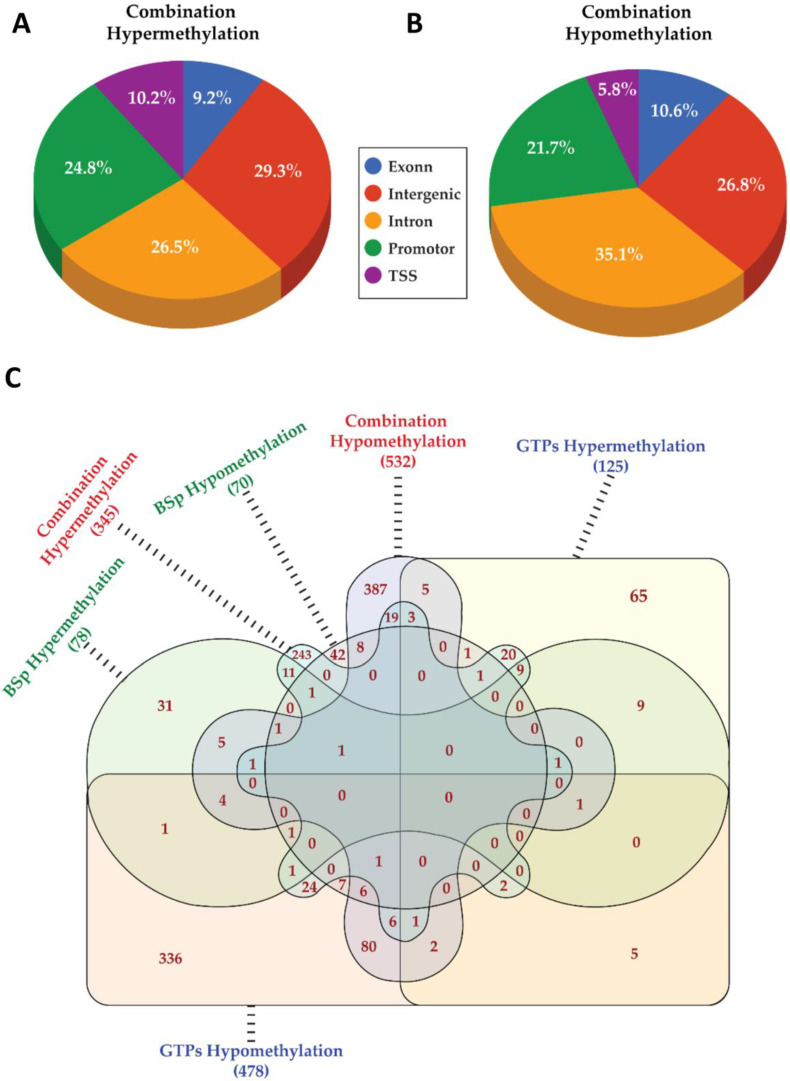
Differential methylation analysis by RRBS across BSp, GTPs and combination treatment group. Pie charts represent genomic distribution of DMGs in (**A**) hypermethylated regions and (**B**) hypomethylated regions in response to combination treatment. (**C**) Venn diagram illustrating a total (unique and overlapping) number of hypomethylated or hypomethylated genes across different dietary treatment groups.

We applied downstream analyses to identify the CpGs methylation levels across the BSp, GTPs and combination treatment groups. A total of 162 and 636 differentially methylated genes (DMGs) were identified (*p*-value ≤ 0.05) in the BSp and GTPs treatment group, respectively. Out of 162 identified DMGs in the BSp treatment group, 77 DMGs were hypomethylated and 85 were hypermethylated. Of 636 identified DMGs in the GTPs group, 503 DMGs were hypomethylated and 133 were hypermethylated. These DMGs distributed amongst the various genomic regions across each treatment group are provided in Supplementary Figure S5A,B (BSp treatment) and Supplementary Figure S6A,B (GTPs treatment). Overall, the combinatorial treatment displayed a higher number of DMGs (996 DMGs), among which 603 DMGs were hypomethylated and 393 were hypermethylated. Additionally, a comprehensive list of the DM transcripts in the different groups is provided in Supplementary File S2: Tables S7–S12. Compared to the BSp or GTPs treatment alone, the combination treatment group showed more variation in the methylation levels with a total of 996 DMGs distributed in various genomic regions (Figure 6A,B). Each of these DMGs in the individual treatment groups had many unique ones overlapping amongst them (Figure 6C). The unique list of DMGs across the genome in each treatment group served as a reference for identifying the correlation between the gene transcription and DNA methylation. 

### 3.7. Integrative Analysis of Transcriptomic and Methylomic Data

**Figure 7:**
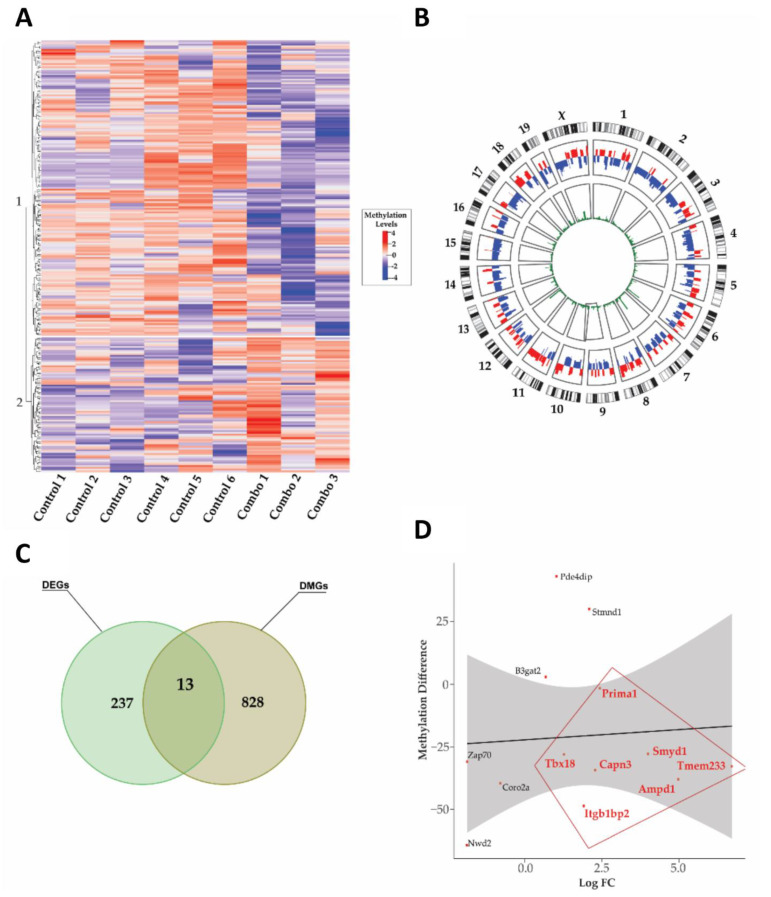
Methylomic-level changes in the combination treatment group. (**A**) Heatmap representing DMGs in control and combination (BSp + GTPs) treatment groups, wherein rows correspond to DMGs, and columns represent biological replicates in control (N_Control_ = 6) and the combination (N_Combination_ = 3) treatment groups. Blue color represents lower methylation level and red indicates higher methylation level. (**B**) Circos plot showing the overall distribution of DMGs across chromosomes. Y chromosomes were excluded as only female mice were used in this study. (**C**) Integrated analysis identified 13 target genes showing both differentially methylated (DM) and differentially expressed (DE) in the combination treatment group. The green circle represents DEGs; the yellow color represents DMGs. (**D**) Scatter plot showed in 13 target genes, 7 genes (*Ampd1*, *Capn3*, *Itgb1bp2*, *Prima1*, *Tbx18* and *Tmem233*, dots in red color) were upregulated and hypomethylated. The *y*-axis represents methylation difference, and the *x*-axis represents log_2_FC.

To better visualize the methylomic-level changes across the different treatment groups, a heatmap was generated across the control-combination (Figure 7A), control-BSp (Supplementary Figure S7A) and control-GTPs treatment groups (Supplementary Figure S7B). Additionally, we also examined the DNA methylation pattern changes across the chromosomes in the combination treatment group using Circos plotting (Figure 7B). To further determine the potential role of DNA methylation on gene expression, we applied an integrated analysis by analyzing the DMGs obtained from the RRBS analysis and the corresponding DEGs in the different treatment groups. In the BSp and GTPs treatment groups, six target genes were identified, which showed as both differentially methylated and expressed (BSp_DEGs+DMGs_ =4 and GTPs_DEGs+DMGs_ =2) (Supplementary Figure S8A,B). The combination treatment group exhibited a higher correlation among the DEGs and DMGs with a total of 13 identified target genes (*Ampd1*, *B3gat2*, *Capn3*, *Coro2a*, *Itgb1bp2*, *Nwd2*, *Pde4dip*, *Prima1*, *Stmnd1*, *Symd1*, *Tbx18*, *Tmem233* and *Zap70*) (Figure 7C). To better envisage the association of the DNA methylation and gene transcription among these 13 identified target transcripts, we generated a scatter plot between the methylation difference and gene expression changes (Figure 7D). Out of 13 transcripts, seven genes (highlighted in Figure 7D) followed a canonical trend between the gene transcription and DNA methylation (gene upregulation is correlated with DNA hypomethylated, and vice versa).

Because our previous study on the Her2/neu mouse model revealed that combinatorial treatment had a more significant impact on methylation changes than BSp and GTPs treatment alone, we therefore compared the methylomic profile in an SV40 mouse to that in a Her2/neu mouse model [8]. As a result, we identified 33 overlapping DMGs in both SV40 and Her2/neu mice by BSp treatment, 248 overlapping DMGs in the GTPs group and 266 overlapping DMGs in the combination group (Supplementary File S3: Tables S13–S15). The overlapped DMGs number (*n* = 266) is much higher than the overlapped DEGs (*n* = 2) in the combination group across the two different transgenic mouse models that showed similar responses to the combination treatment in inhibiting BC, suggesting consistent epigenetic landmark changes may respond to this dietary treatment regardless of the genotypic difference. In summary, the combination treatment group showed a more significant impact on both the transcriptomic and methylomic levels in two different mouse models, indicating that epigenetic mechanism-induced gene expression changes may play a role in the regulation of the preventive effects of the combination treatment of BSp and GTPs in inhibiting BC. 

### 3.8. Biological Functions and Pathways Affected by Combinatorial Treatment in SV40 Mice at DNA Methylation Level

**Figure 8:**
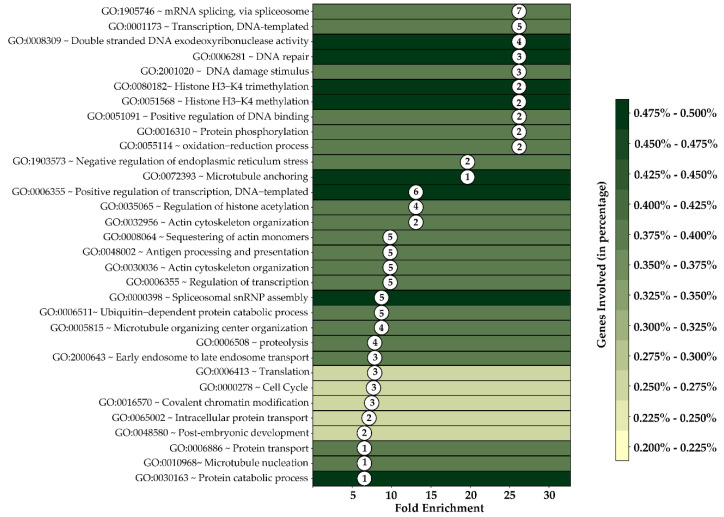
Gene function association by gene ontology (GO) analysis with DAVID in combination treatment group. *Y*-axis represents multiple cellular pathways associated with combination treatment group, and *X*-axis represents fold enrichments. Each GO term comprises the total number of significant DMGs that were described in circles inside the plot. Color code is based on the total number of individual GO terms estimated in percentage.

To better understand the biological functions of DNA methylation changes by combination treatment, we analyzed the functional gene associations in significantly altered methylomic profiles using DAVID. Our results indicated that DMGs-involved multiple cellular pathways were regulated by the combination dietary treatment of BSp and GTPs, such as DNA repair, oxidative phosphorylation, DNA methylation, histone acetylation, covalent chromatin modifications, apoptosis, the cell cycle and many others (Figure 8). These pathway changes due to methylation profiling changes in the relevant genes may significantly contribute to the combinatorial dietary regimen-induced BC inhibitory effects. This result suggests that consuming these dietary botanicals in combination can reinforce the anticancer benefits by causing changes in the methylomic level of the key genes that affect important biological pathways. 

### 3.9. Effects of BSp, GTPs and Combination Treatment on Global Epigenetic Profiles

**Figure 9:**
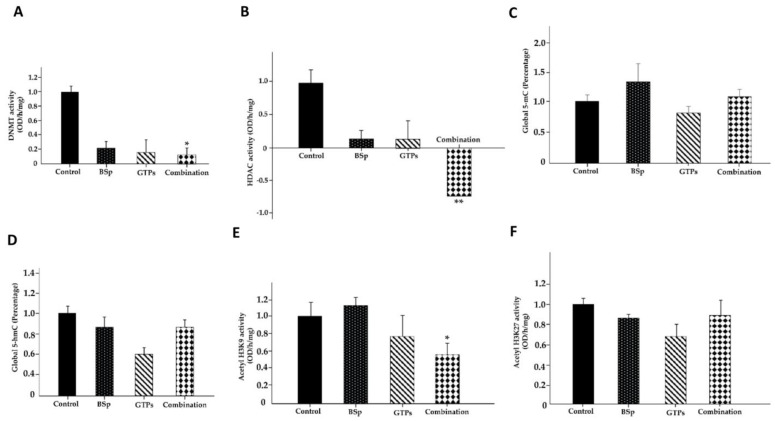
Global epigenetic profiles in response to BSp, GTPs and combination treatment groups. (**A**) DNMT activity. (**B**) HDAC activity. (**C**) 5-mC level. (**D**) 5-hmC level. (**E**) Acetyl-H3K9 activity. (**F**) Acetyl-H3K27 activity across different treatment groups. Columns, mean; bars, SE; * *p* < 0.05, significantly different from the control group.

We further evaluated the epigenetic-driven mechanisms due to the administration of different dietary groups. Figure 9A,B showed a significant decrease in the enzymatic activities of the DNMTs and HDACs in the combination treatment group, implying that the administration of BSp and GTPs in combination may lead to lower levels of global DNA methylation and higher levels of histone acetylation in an SV40 mouse model. We also investigated the global DNA methylation by detecting the 5-methylcytosine (5-mC) content and DNA hydroxymethylation (5-hmC) status in SV40 mammary tumors. Unlike the DNMTs and HDAC activities, the combination treatment had a marginal impact on the 5-mC and 5-hmC levels compared to the singly administered groups (Figure 9C,D). We also evaluated the effect of BSp, GTPs and the combination treatment on the acetyl histone3-lysine9 (H3-K9) activity and acetyl histone3-lysine27 (H3-K27) activity (Figure 9E,F). As a result, the combination treatment led to a significant decrease in the H3K9 residual activity but not in the H3-K27 activity. Overall, these findings suggest that the combination treatment appears to induce more obvious epigenetic changes in the DNMT, HDAC and acetyl H3K9 enzymatic activities than the singly administered groups. This may result in global demethylation and an increased acetylation status leading to gene transcriptional activation such as tumor suppressor genes, which may further explain the chemopreventive effects of the combination treatment on BC. 

## 4. Discussion

Clinical trials have demonstrated that cruciferous vegetable BSp and green tea as well as their phytochemical extracts, including SFN and GTPs, are highly effective therapeutic and chemopreventive agents against various cancer types [8,14,16,18]. Importantly, these botanicals are considered as an “epigenetics diet” that can regulate key epigenetic pathways, contributing to their cancer inhibitory effects. According to our previous studies, BSp and GTPs can be used as therapeutic or preventative agents against BC when administered singly or in combination [8,35,36,37]. Numerous studies on bioactive vegetables have demonstrated their efficacy, safety, pharmacokinetics and molecular processes. However, there is still limited knowledge of the underlying causes, such as how these dietary botanicals affect transcriptomic and DNA methylation profiling, contributing to their chemopreventive effects.

In this study, we applied a genome-wide analysis to the multi-omics data and explored the underlying mechanisms by BSp and GTPs treatment (alone and in combination) in the BC transgenic SV40 mouse model. This multi-omics approach integrates transcriptomic and methylomic data that facilitate understanding how epigenetic mechanisms can influence gene transcriptional profiling in a genome-wide perspective in response to BSp and/or GTPs dietary administration. We categorized the global DNA methylation and gene expression patterns in mouse mammary tumors from different treatment groups. Our results demonstrated that exposure to BSp, GTPs and the combination treatment (BSp + GTPs) led to changes in the transcriptome and genome-wide DNA methylation profiles across different genes. We identified that the combination treatment exhibited a greater efficacy in inhibiting tumor growth than the single treatment groups. Consistently with the phenotypic trend across different dietary groups, our results also showed that the combination treatment exhibited a more significant impact in modulating the transcriptomic and methylomic profiles than that of any single treatment. This could potentially serve as a critical contributor toward combinatorial approach-induced preventive effects on BC.

Our transcriptomic analysis in the SV40 transgenic mouse model identified 250 DEGs in the combination group compared to the BSp and GTPs treatment group with fewer DEGs. Our previous study on the Her2/neu transgenic mouse model reported 895 DEGs in the combination treatment group with even lesser DEGs in the BSp or GTPS treatment groups [8]. Upon comparing the DEGs in two different breast cancer mouse models, we identified two common DEGs that showed as upregulated in the combination treatment group (*Cbl* and *Zfp800*) in both mouse models, indicating universal impacts on these genes by combinatorial treatment across the different mouse models. In contrast to the combination treatment group, BSp or GTPs individually did not have overlapping DEGs at transcriptomic levels between the two mouse models. *Cbl* is a proto-oncogene with E3 ubiquitin-protein ligase activity that is primarily responsible for signal transduction in response to various kinds of stimuli [38,39]. The primary function of *Cbl* is to ubiquitously activate RTKs, thereby suppressing the RTK signaling toward lysosomal degradation [40]. Studies have shown that *Cbl* can also act as a tumor suppressor gene involved in the pathogenesis of different cancer types. For instance, a study demonstrated the primary function of *Cbl* in restricting tumor cell proliferation and invasion [41,42]. Another study in human BC tissues showed that the overexpression of the *Cbl* gene led to malignant behaviors by directly targeting microRNA (miRNA) miR-124-3p functions [34]. Similarly, a study reported that increased *Zfp800* gene expression can inhibit tumor cell proliferation in pancreatic cancer [43]. This study also reported an association of *Zfp800* with various biological pathways, such as cell proliferation, cell growth, etc. Thus, the identification of these two genes that were significantly differentially expressed in both mouse models may provide mechanistic insights into how the combination treatment of BSp and GTPs exhibited the most prominent chemopreventive effects on breast cancer than any single treatment. *Cbl* and *Zfp800* and their related pathways could be target responders to this novel combination treatment and may contribute to its preventive effects against BC. These effects may be universal and independent of the tumor genotype. It is possible that each preclinical model might influence different genes to target the same signaling or metabolic pathways leading to similar outcomes. For example, both models regulate important biological pathways such as DNA repair, the cell cycle, protein transportation and histone acetylation as well as others. The regulation of these important signaling pathways may contribute to the preventive effects of the combination strategy against BC.

Aligning with our previous study [8], our methylomic-level analysis also revealed that the combination treatment led to a more significant number of DMGs in comparison to the BSp and GTPs alone in the SV40 model. Overall, the combined treatment resulted in 250 DEGs and 996 DMGs compared to the control. The inconsistent magnitude of the numbers of DEGs and DMGs is most likely because several DMGs can reflect one gene as the significantly altered DNA methylation loci can distribute in different regions of the same gene via the RRBS method, whereas DEGs can be uniquely identified through RNA sequencing. Simultaneously, our integrated analysis revealed 13 target genes that showed as both significantly differentially expressed and methylated in response to the combination treatment group. Out of these 13 transcripts, seven genes (*Ampd1, Capn3, Itgp1bp2, Prima1, Tmem233 and Symd1*) followed a positive relationship between the DNA methylation and gene transcription regulation (DNA hypomethylation leads to gene upregulation, and vice versa). As these gene changes may result in alterations of multiple key cellular pathways, we therefore presume that the combinatorial treatment of BSp and GTPs may reverse aberrant epigenetic landmarks leading to altered key gene expression profiles. Although only 13 genes are identified that may be regulated by DNA methylation, this suggests that other mechanisms may participate in combination treatment-induced gene expression changes beyond epigenetic regulation, and an increased sample size in future studies will help identify more target genes modulated through epigenetic mechanisms. In concordance with our above findings, our study also revealed the combination treatment had a more significant impact on several essential epigenetic modulators, such as the DNMTs, HDAC and acetyl H3-K9 enzymatic activities, than any single treatment of BSp or GTPs in the SV40 model. For example, we found the combinatorial treatment reduced the global DNMT enzymatic activity. This might explain that the majority of the identified DMGs in the combination group were hypomethylated. However, the global cytosine methylation (5-mC) levels remain constant. This discrepancy between DNMT and 5-mC has previously been shown in human prostate cancer, which is associated with the tumor stage and differentiation that can be used as a biomarker for prostate cancer [44].

Overall, our studies indicate that the dietary administration of combined BSp and GTPs can induce more significant impacts on BC suppression than any of these nutrients administered alone, which may be due to a possible additive or synergistic impact of this novel dietary regimen on transcriptomic and methylomic profiling across different transgenic BC mouse models. As a result, our research could lead to a novel dietary approach in the prevention of BC and also help the identification of biomarkers in response to this combinatorial intervention.

## Figures and Tables

**Table 1:** The primer sequence for each specific genes of interest.

Gene	Forward Primer Sequence (5′-3′)	Reverse Primer Sequence (5′-3′)
**Common DEGs in SV40 and Her2/neu Mouse Models**		
*Cbl*	CGGTAATTGTTGCGTTTCCA	ACAGCTC-GCTCCCGAAGAA
*Zfp800*	CTCGTGACCACCTTCGGCTA	TCCTGGACGTAGCCTTCGGT

**Table 2:** Total number of DEGs across the different treatment groups.

DEGs	BSp vs. Control	GTPs vs. Control	Combination vs. Control
Upregulated	119	30	225
Down-regulated	74	49	25
NOT significant	14,573	14,717	14,516
**TOTAL**	**14,766**	**14,766**	**14,766**

**Table 3:** Top 20 upregulated and down-regulated genes with the combination treatment, ranked by fold change.

Gene Symbol	Gene Expression Fold Change (log_2_FC)	Average Differential Expression	*p* Value for Differential Expression	False Discovery Rate (FDR)	Significance
*Myh4*	10.422	0.712	2.15 × 10^−7^	1.51 × 10^−4^	Upregulated
*Pvalb*	9.114	−1.467	7.09 × 10^−5^	1.06 × 10^−2^	Upregulated
*Myl1*	8.349	0.304	3.16 × 10^−4^	3.26 × 10^−2^	Upregulated
*Mybpc1*	8.231	−1.935	7.09 × 10^−7^	3.10 × 10^−4^	Upregulated
*Art1*	8.173	−3.221	3.22 × 10^−8^	3.65 × 10^−5^	Upregulated
*Mstn*	8.102	−3.953	4.94 × 10^−10^	1.83 × 10^−6^	Upregulated
*Actn3*	7.628	−0.250	4.23 × 10^−8^	4.46 × 10^−5^	Upregulated
*Tnnc2*	7.480	1.025	3.57 × 10^−5^	6.35 × 10^−3^	Upregulated
*Tnnt3*	7.306	2.088	5.45 × 10^−5^	8.85 × 10^−3^	Upregulated
*Cacna1s*	7.299	−2.823	8.35 × 10^−5^	1.16 × 10^−2^	Upregulated
*Atp2a1*	7.167	2.905	2.80 × 10^−5^	5.56 × 10^−3^	Upregulated
*Rps27rt*	−5.578	−1.521	2.88 × 10^−4^	3.11 × 10^−2^	Down-regulated
*Pla2g4b*	−4.945	0.970	1.12 × 10^−5^	2.67 × 10^−3^	Down-regulated
*Gm29106*	−2.796	−2.370	7.83 × 10^−4^	5.90 × 10^−2^	Down-regulated
*Boll*	−2.784	−3.542	2.28 × 10^−7^	1.53 × 10^−4^	Down-regulated
*Myh6*	−2.563	−2.208	1.73 × 10^−4^	2.13 × 10^−2^	Down-regulated
*Gm5796*	−2.484	−4.942	6.41 × 10^−6^	1.76 × 10^−3^	Down-regulated
*Olfr1344*	−2.294	−4.031	2.15 × 10^−4^	2.54 × 10^−2^	Down-regulated
*Gm36368*	−2.204	−3.215	5.79 × 10^−4^	5.00 × 10^−2^	Down-regulated
*Csl*	−2.191	−3.338	3.53 × 10^−5^	6.35 × 10^−3^	Down-regulated

## Data Availability

All relevant data are within the paper and its Supplementary Materials files.

## Supplementary Materials

The following supporting information can be downloaded at: https://www.mdpi.com/article/10.3390/cells12010014/s1. The Supplementary Materials include Supplementary Figures; Supplementary File List; Supplementary File S1: Transcriptomic level changes in BSp, GTPs and Combo treatment; Supplementary File S2: Global methylation level changes in BSp, GTPs and Combo treatment; Supplementary File S3: Methylation level changes across two different mouse models.

## Abbreviations

BSp	Broccoli sprouts
BC	Breast cancer
Combo	Combination
GTPs	Green tea polyphenols
DAVID	Database for Annotation, Visualization and Integrated Discovery
DMGs	Differentially methylated genes
DEGs	Differentially expressed genes
DMRs	Differentially methylated regions
DNMTs	DNA methyltransferases
DNA	Deoxyribonucleic acid
EGCG	Epigallocatechin gallate
ER(-) BC	Estrogen receptor-negative breast cancer
FC	Fold change
FDR	False discovery rate
GO	Gene ontology
HDACs	Histone deacetylases
mm	Mus musculus
qRT-PCR	Quantitative reverse transcription PCR
QC	Quality control
PCA	Principal component analyses
RNA	Ribonucleic acid
RNA-seq	RNA sequencing
RRBS	Reduced representation bisulfite sequencing
