# Supplementary material for: Genome-Wide Analysis on Transcriptome and Methylome in Prevention of Mammary Tumor Induced by Early Life Combined Botanicals
Source: Cells. 2022 Dec 21;12(1):14. doi: 10.3390/cells12010014 (PMC9818885; doi:10.3390/cells12010014)
Supplement: Supplementary file 1 [file cells-12-00014-s001.zip › Supplementary Figures.pdf]

Supplementary Figures

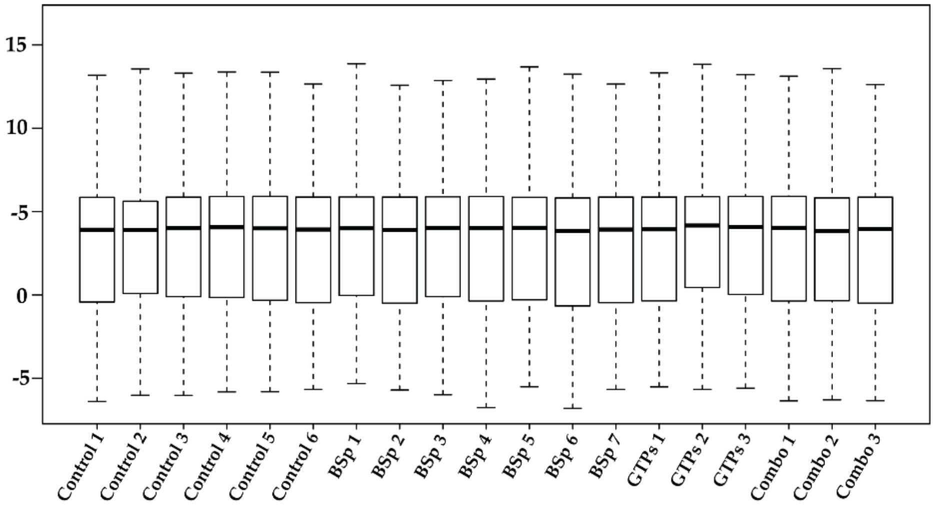

Figure S1. Boxplot illustrating sample counts across different treatment group ($N_{Control}$ = 6, N_{BSp} = 7, N_{GTPs} = 3, $N_{Combination}$ = 3) with no sample outliers.

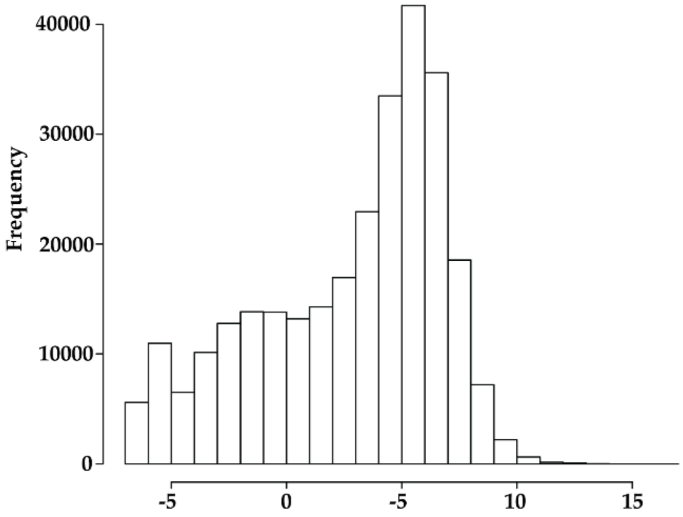

Figure S2. Histogram demonstrating normal distribution of total feature counts across different samples. Y-axis represents the total frequency across 19 samples and X-represents total feature by counts.

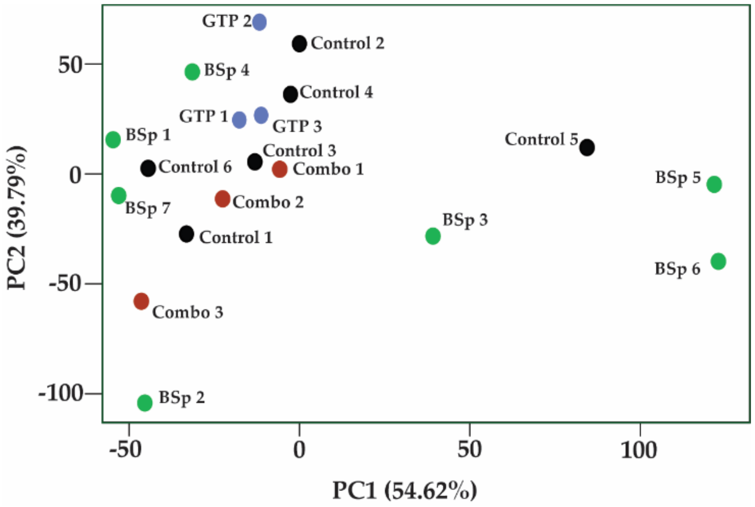

Figure S3. Normalized PCA across PC1 (54.62%) v/s PC2 (39.79%) using BSp, GTPs and Combination (BSp + GTPs) induced differentially expressed genes (DEGs).

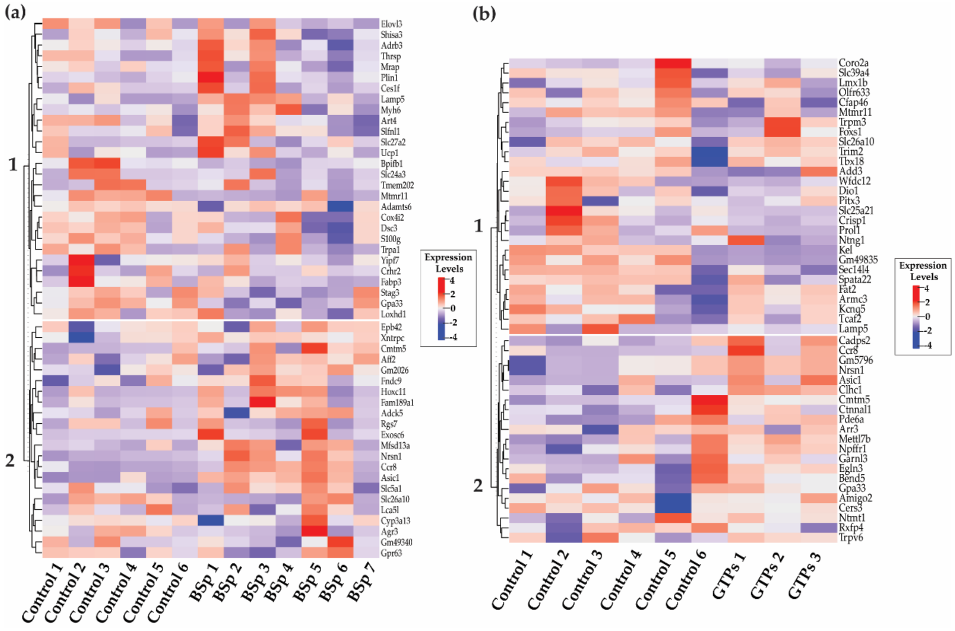

Figure S4. Heatmap representing the top 50 DEGs in **(a)** BSp treatment group and **(b)** GTPs treatment group based on *p* value. Each row corresponds to differentially expressed transcripts and each column represents biological replicates in control ($N_{Control} = 6$), BSp ($N_{BSp} = 7$) and GTPs ($N_{GTPs} = 3$) treatment group.

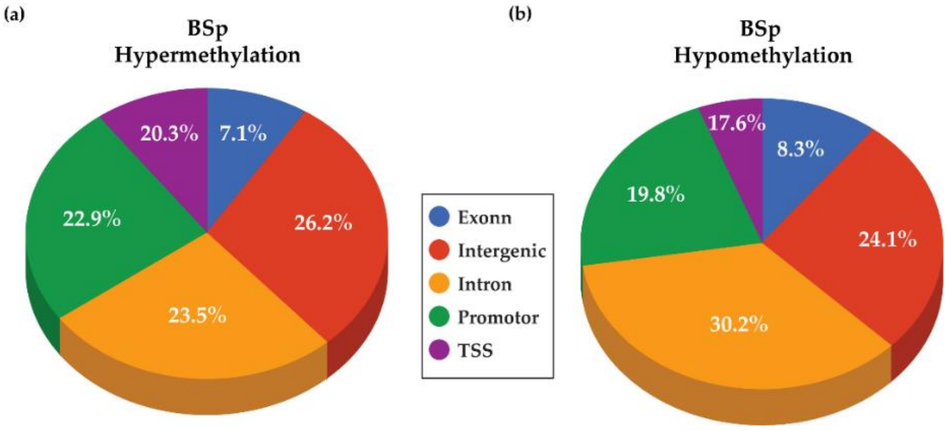

Figure S5. Pie charts representing genomic distribution of differentially methylated genes (DMGs) by BSp treatment in (**a**) hypermethylated regions and **(b)** hypomethylated regions.

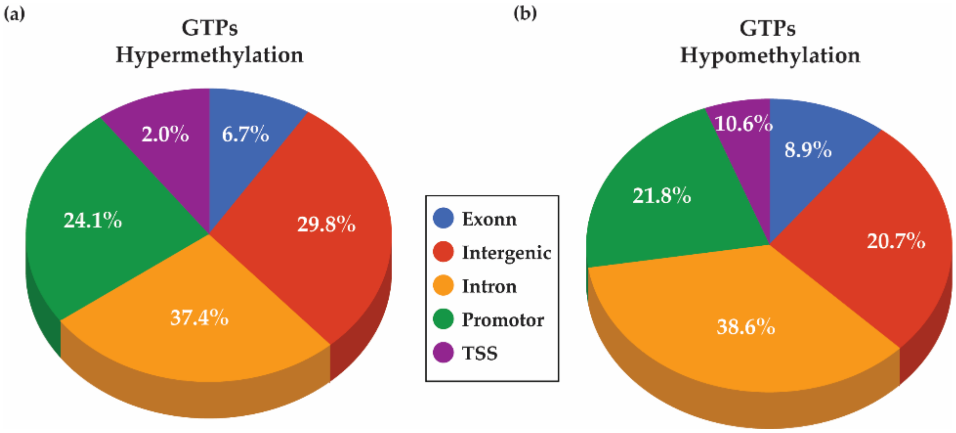

Figure S6. Pie charts representing genomic distribution of DMGs by GTPs treatment in (**a**) hypermethylated regions and **(b)** hypomethylated regions.

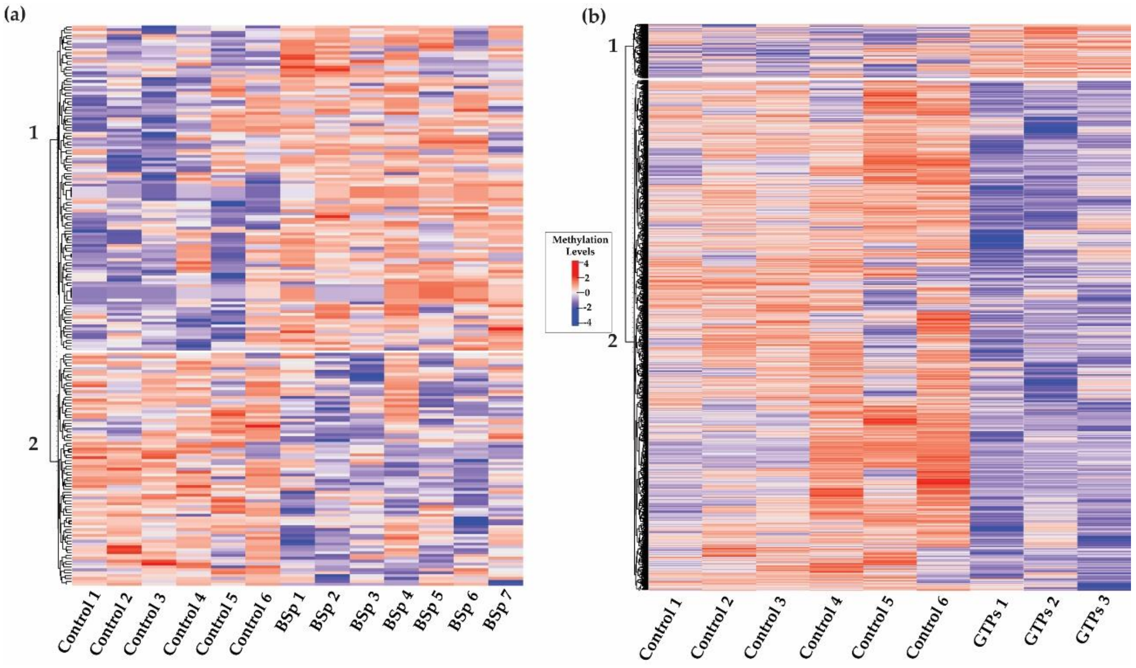

Figure S7. Heatmap representing DMGs in **(a)** BSp treatment group and **(b)** GTPs treatment group based on q value. Each row corresponds to differentially expressed transcripts and each column represents biological replicates in control ($N_{Control} = 6$), BSp ($N_{BSp} = 7$) and GTPs ($N_{GTPs} = 3$) treatment group across two heatmap clusters (1 and 2).

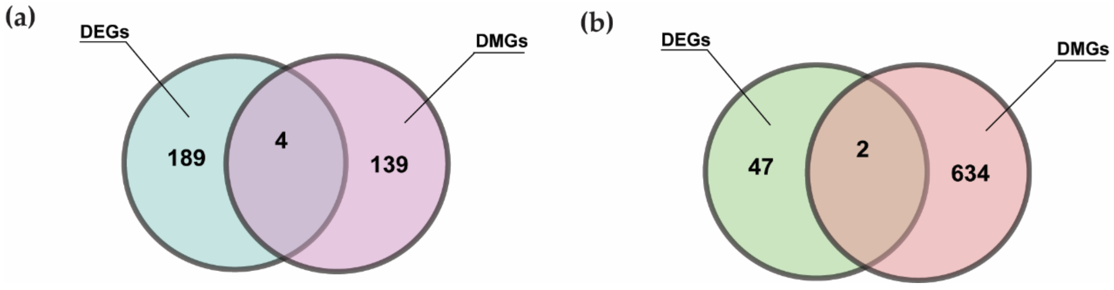

Figure S8. Venn diagram representing unique DEGs and DMGs in **(a)** BSp treatment group and **(b)** GTPs treatment group.
