# Supplementary material for: Genome-Wide Analysis on Transcriptome and Methylome in Prevention of Mammary Tumor Induced by Early Life Combined Botanicals
Source: Cells. 2022 Dec 21;12(1):14. doi: 10.3390/cells12010014 (PMC9818885; doi:10.3390/cells12010014)
Supplement: Supplementary file 1 [file cells-12-00014-s001.zip › Supplementary File List.pdf]

Supplementary File List

Supplementary File1: Table S1. Reference list of genes with P-value and fold change in BSp treatment group.

Supplementary File1: Table S2. A list of differentially expressed (DE) genes in BSp treatment ($p<0.05$) and calculated fold change at transcriptome level.

Supplementary File1: Table S3. Reference list of genes with P-value and fold change in GTPs treatment group.

Supplementary File1: Table S4. A list of differentially expressed (DE) genes in GTPs treatment ($p<0.05$) and calculated fold change at transcriptome level.

Supplementary File1: Table S5. Reference list of genes with P-value and fold change combination (BSp + GTPs) treatment group.

Supplementary File1: Table S6. A list of differentially expressed (DE) genes in the combination (BSp + GTPs) treatment group (P-value<0.05) and calculated fold change at transcriptome level.

Supplementary File2: Table S7. A list of nineteen single-end libraries (N_{Ctrl} = 6, N_{BSp} = 7, N_{GTPs} = 3, and $N_{Combination}$ = 3) wherein each libraries produced a minimum of 7 Gb clean reads, which were sequenced and aligned to the reads to mouse reference NCBI GRCm39 genome using Bismark. The reads of individual samples were mapped to the reference genome, thereby generating relevant BAM files for different samples within each group with an average of ~68.78% of reads were aligned uniquely to the reference genome, with a mean Phred score of 34.20.

Supplementary File2: Table S8. List of genes that are differentially methylated (DM) in BSp treatment group.

Supplementary File2: Table S9. List of genes that are differentially methylated (methylation difference) in GTPs treatment group.

Supplementary File2: Table S10. Unique genes that are differentially expressed (P-value<0.05) and differentially methylated (methylation difference) in BSp and GTPs treatment group.

Supplementary File2: Table S11. Reference list of methylation level changes in the combination (BSp + GTPs) treatment group.

Supplementary File2: Table S12. Unique differentially expressed genes (P-value<0.05) and differentially methylated (methylation difference) in the combination (BSp + GTPs) treatment group.

Supplementary File3: Table S13. Genes that were differentially methylated (methylation difference) across SV40 and Her2/neu mice models in BSp treatment group.

Supplementary File3: Table S14. Genes that were differentially methylated (methylation difference) across SV40 and Her2/neu mice models in GTPs treatment group.

Supplementary File3: Table S15. Genes that were differentially methylated (methylation difference) across SV40 and Her2/neu mice models in the combination (BSp + GTPs) treatment group.
